# Mitochondria: An Emerging Unavoidable Link in the Pathogenesis of Periodontitis Caused by *Porphyromonas gingivalis*

**DOI:** 10.3390/ijms25020737

**Published:** 2024-01-06

**Authors:** Shiyin Luo, Tong Xu, Qifan Zheng, Aijia Jiang, Jiahui Zhao, Yue Ying, Nan Liu, Yaping Pan, Dongmei Zhang

**Affiliations:** 1 Department of Periodontics, School of Stomatology, China Medical University, Shenyang 110002, China; 2022121888@cmu.edu.cn (S.L.); 2022110366@cmu.edu.cn (T.X.); 2022121944@cmu.edu.cn (Q.Z.); 20172238@cmu.edu.cn (A.J.); 2023122032@cmu.edu.cn (J.Z.); 2023121967@cmu.edu.cn (Y.Y.); 2023240171@cmu.edu.cn (N.L.); 2 Department of Periodontics and Oral Biology, School of Stomatology, China Medical University, Shenyang 110002, China; yppan@cmu.edu.cn

**Keywords:** mitochondria, periodontitis, *Porphyromonas gingivalis*, energy metabolism, mitochondrial dysfunction, mitochondrial quality control

## Abstract

*Porphyromonas gingivalis* (*P. gingivalis*) is a key pathogen of periodontitis. Increasing evidence shows that *P. gingivalis* signals to mitochondria in periodontal cells, including gingival epithelial cells, gingival fibroblast cells, immune cells, etc. Mitochondrial dysfunction affects the cellular state and participates in periodontal inflammatory response through the aberrant release of mitochondrial contents. In the current review, it was summarized that *P. gingivalis* induced mitochondrial dysfunction by altering the mitochondrial metabolic state, unbalancing mitochondrial quality control, prompting mitochondrial reactive oxygen species (ROS) production, and regulating mitochondria-mediated apoptosis. This review outlines the impacts of *P. gingivalis* and its virulence factors on the mitochondrial function of periodontal cells and their role in periodontitis.

## 1. Introduction

Periodontitis is defined as a chronic inflammatory disease under the effect of dysbiotic plaque biofilms, leading to the destruction of periodontal tissues, the resorption of alveolar bone, and even the loosening and loss of teeth [1,2]. Since periodontitis is a multifactorial disease, its treatment is constantly being explored and refined. A critical appraisal of authoritative sources proposes that the periodontitis risk assessment should look at nonclinical indicators (e.g., microbiological, immunological, genetic, and epigenetic) in addition to relying on clinical factors [3]. Immunology in periodontitis revealed that neutrophils are early responders in the immunopathologic reaction to subgingival biofilm and attract macrophages, dendritic cells, and T cells to the foci [4]. In the periodontitis process, the struggle between microbial infection and host immune system defense determines the degree and persistence of inflammatory infiltration and the enriched type of immune cells [5]. Moreover, the imbalanced immune homeostasis in periodontitis is believed to be the pathologic basis for its association with systemic diseases such as diabetes [6], obesity [2], and atherosclerotic cardiovascular disease [7]. Of note, it has been affirmed that the disturbances of the host immune response triggered by the dysbiotic microbiome are the underlying pathological mechanisms of low-grade systemic inflammation elicited by periodontitis [8].

Periodontal flora colonizes the subgingival region and transforms from harmless to virulent under certain circumstances, with *Porphyromonas gingivalis* (*P. gingivalis*) considered to be the keystone bacterium. Previous studies have focused on the host cell functional alteration by *P. gingivalis* and its virulence factors at the cellular and molecular levels, and the molecular pathways involved in this inflammatory immune response [9,10,11]. Currently, there is a rising interest from an organelle perspective in exploring the pathology of periodontitis, especially mitochondria, as the important cellular energy production factories driving normal cellular activity.

Govindaraj et al. revealed that 60% of patients with chronic periodontitis have different forms of mitochondrial structural abnormalities in their gingival tissue [12]. Furthermore, according to a complete mitochondrial DNA (mtDNA) sequence analysis, they found the inflamed tissue was more prone to mtDNA mutations and mitochondrial dysfunction [12]. An animal experiment observed that periodontium cells from periodontitis mice displayed a significantly severer level of impaired mitochondrial oxidative phosphorylation function and oxidative stress than the control group [13]. In a vitro study, similar disruptions in mitochondrial structure and function were proven in human gingival fibroblasts (HGFs) from patients with chronic periodontitis [14]. More importantly, the extra stimulation of lipopolysaccharide (LPS) from *P. gingivalis* exacerbated mitochondrial abnormalities in inflamed HGFs and induced these abnormalities in the healthy HGFs, indicating the mitochondrial dysfunction triggered by *P. gingivalis* may play a vital part in the pathogenesis of periodontitis [14]. Also, changes in the functional mitochondrial molecules can influence the state of periodontal cells. For example, abnormal enhancement of mitochondrial permeability transition pores was found to aggravate the pro-inflammatory activation of macrophages [15].

Additionally, mitochondrial dysfunction may be a shared pathogenic background in periodontal comorbidities. A cross-sectional study demonstrated that a mitochondrion-derived biomarker of mitochondrial dysfunction, the blood sample concentration of methylmalonic acid, may act as a bridging indicator in the association between periodontitis and cognitive impairment in older adults aged ≥60 years [16]. Similarly, mitochondrial oxidative deregulation was proved to be a mutual systemic cytopathological feature of periodontitis and type 2 diabetes [17,18]. Moreover, there is evidence to suggest that the application of prominent polyphenolic compounds that optimize mitochondrial function, such as resveratrol, ameliorated the destruction of renal structures in mice with periodontitis [19] and alleviated the ferroptosis of alveolar osteocytes in mice with diabetic periodontitis [20]. In light of the mitochondrial pathway being so extensively involved in the pathological process of periodontitis, it has received mounting interest in exploring how the crucial periodontal pathogen, *P. gingivalis*, disrupts mitochondria.

The effect of *P. gingivalis* on specific functional mitochondrial molecules varies from its distinct virulence factors. And how these mitochondrial changes caused by *P. gingivalis* are involved in the periodontal inflammatory process is not yet clear. In this review, we presented how mitochondrial metabolic transformations and mitochondrial signaling molecules, such as mitochondrial DNA, mitochondrial ROS, and mitochondrial-mediated apoptotic effectors, were involved in periodontitis. In addition, mitochondrial dysfunction caused by *P. gingivalis* was described and summarized in an attempt to better understand how *P. gingivalis* destabilizes periodontal homeostasis.

## 2. *Porphyromonas gingivalis* and Periodontitis

*P. gingivalis* is a gram-negative anaerobe mainly existing in the subgingival biofilms. The diverse virulence factors of *P. gingivalis*, including LPS, fimbriae, gingipain, capsules, etc., act as adhesive or destructive agents independently or synergistically mediating the periodontal inflammation and periodontal tissue damage [9]. *P. gingivalis* causes extensive destruction to various cells in periodontal tissue, with pro-inflammatory response as the main manifestation. *P. gingivalis* adheres to and invades cells through the collaboration of different virulence factors, perturbing the structural and functional integrity of the gingival epithelium, the first defensive line of periodontium [21]. *P. gingivalis* also penetrates the epithelium thus attacking the deeper cells of the periodontal tissue. Under *P. gingivalis* infection, gingival fibroblasts, as the essential components of the periodontium, undergo a shift to a pro-inflammatory phenotype, recruiting leukocytes, secreting protein hydrolysing enzymes, and inducing osteoclast formation [22]. It can directly induce monocyte/macrophage differentiation into osteoclasts through the receptor activator of the nuclear factor-kappa B (NF-κB) ligand pathway, thus promoting the absorption of the bone mineral matrix and collagen matrix [23]. Meanwhile, *P. gingivalis* and its virulence factors prevent osteoblast differentiation and bone formation [24,25]. More importantly, *P. gingivalis* elicits a massive and widespread release of pro-inflammatory factors from immune cells [26,27] and promotes the expansion of immune-suppressive cells [28] as a way to expand its inflammatory damaging and immune evasion effects. When pro-inflammatory cytokines, such as interleukin (IL)-1, -6, -11,-17, and tumor necrosis factor-alpha (TNF-α), achieve critical concentrations, the inflammatory response leads to periodontal tissue damage [29]. It is worth mentioning that in addition to its local effects in the periodontal tissues, *P. gingivalis* can, in some cases, invade distant organs through haematogenous dissemination, causing damage to other tissue [30]. In a word, *P. gingivalis* interferes with multiple periodontal cells.

## 3. Mitochondrial Involvement in Periodontal Inflammation

### 3.1. Mitochondrial Structure and Function

As central regulators of aerobic energy production, mitochondria rely primarily on electron transfer and proton gradients which occur across the mitochondrial membrane to influence cellular homeostasis, generating sufficient ATP in the process. Mitochondria are double membrane organelles. In the mitochondrial matrix, the mtDNA is present in multiple copies and packed into compact particles, termed nucleoids. The outer mitochondrial membrane (OMM) separates the mitochondria from the cytosol and is generally permeable serving as a platform for communication between signaling molecules. The inner mitochondrial membrane (IMM) delimits the mitochondrial matrix and folds inward into deeply convoluted cristae for expanding the surface to ensure that mitochondrial electron transport chain (ETC) complexes attached to the membrane can conform to the needs of cellular energy production. And the ETC complexes contain NADH-ubiquinone oxidoreductase (complex I), succinate-ubiquinone oxidoreductase (complex II), ubiquinol-cytochrome c reductase (complex III), cytochrome c oxidase (complex IV), as well as the FoF1-ATP synthase (ATP synthase or complex V) and two mobile electron carriers, coenzyme Q (CoQ) and cytochrome c (cyt c) [31]. Compared to the OMM, the IMM is impermeable to support the transmembrane proton gradient and therefore requires a specific mitochondrial carrier family to transport solutes [32]. When subjected to stress or injury, the mitochondrial structure and the location and function of mitochondrial proteins shift. These changes result in aberrant alterations in mitochondrial molecules and shifts in mitochondrial metabolic types, thus affecting the phenotype of immune cells and taking part in the inflammatory response.

### 3.2. Mitochondria Are Involved in the Inflammatory Response

Mitochondrial ROS (mtROS), efflux of mitochondrial contents, and mitochondria-dependent apoptotic molecules are involved in the activation of inflammatory pathways. The activation of inflammasome and polarization of immune cells correlate with mtROS. mtROS mainly includes oxygen radicals that arise from the excessive electrons transferring to oxygen during mitochondrial respiration which can bind to lipids, proteins, and DNA, causing oxidative damage and corresponding molecular dysfunction [33]. Studies have confirmed that mtROS interacted directly with NOD-like receptor thermal protein domain associated protein 3 (NLRP3), triggering its activation [34,35]. Although mitochondria are not the only source responsible for ROS production, evidence has confirmed that mitochondria-targeted ROS inhibitors attenuate the NLRP3 inflammatory response more effectively than pan-ROS inhibitors [36]. In addition, mtROS are engaged in the macrophage polarization towards a pro-inflammatory MI phenotype and impair lysosomal function [37].

As an instrumental and fragile functional mitochondrial molecule, mtDNA is susceptible to mtROS and acts as an endogenous inflammatory stimulus. Numerous mitochondrial constituents and metabolites can function as damage-associated molecular patterns (DAMPs) to promote inflammation when released into the cytosol or extracellular milieu. Above all of the DAMPs, mtDNA is one of the most widely and valuably studied. Upon cellular stress or mitochondrial insult, mtDNA can be released into the cytoplasm via three main pathways responsible for the regulation of the permeability of mitochondrial membrane, including Bax/Bak, the voltage-dependent anion channel oligomers, and the mitochondrial permeability transition pore (mPTP) [38]. In the cytoplasm, mtDNA can be detected by multiple pattern recognition receptors, including NLRPs, toll-like receptors (TLRs), and the cyclic GMP/AMP synthase–stimulator of interferon genes systems, triggering aberrant pro-inflammatory and type I interferon (IFN) responses [38]. Since mtDNA is spatially close to the mitochondrial ETC, it is vulnerable to oxidative attack. The oxidized mtDNA turns into fragmentation and generates a specific NLRP3 ligand, ultimately promoting an increase in inflammatory mediators, including IL-1β, IL-18, and IFN [39]. Another study exploring the relationship between mtDNA and NLRP3 pointed out that newly synthesized mtDNAs were essential activators of macrophage NLRP3, which correlated with the fact that they had not yet been packaged by histones to form stable nucleoids and thus are highly susceptible to oxidative damage [40].

Furthermore, mitochondria-mediated apoptosis was revealed as a player in microbial pathogen-triggered inflammation [41]. The activated Bax and Bak pores on the OMM lead to the release of mitochondrial pro-apoptotic factors and the subsequent cell death, whereas the anti-apoptotic effector B cell lymphoma 2 (Bcl-2) serves as a ruling checkpoint during the process [42]. Mitochondria-dependent apoptosis of osteoblasts and human gingival epithelial cells can be initiated under inflammatory factors stimulation, such as cytokines IFN-γ, TNF-α, and transforming growth factor β1 [43,44]. However, the apoptotic process appears oppositely in the real periodontitis condition. Bulut et al. revealed that a high rate of positive expression of Bcl-2 has been reported in the inflammatory gingival tissues of patients with generalized aggressive periodontitis [45]. Similarly, up-regulation of anti-apoptosis member genes of oral neutrophils was identified in patients with chronic periodontitis compared with healthy counterparts by examining the transcriptome [46], indicating that delayed apoptosis may be behind the localized retention and accumulation of inflammatory cells, resulting in progressive periodontal destruction.

### 3.3. Mitochondrial Energy Metabolism Shifts with Periodontal Condition

Cellular respiration comprises glycolysis in the cytoplasm, mitochondrial tricarboxylic acid (TCA) cycle, and mitochondrial oxidative phosphorylation (OXPHOS). OXPHOS occurring in the ETC utilizes the reducing equivalents generated by the TCA cycle to transfer electrons to O_2_, forming H_2_O and actuating the transmembrane movement of protons to power the synthesis of ATP, thereby completing oxidative phosphorylation and producing sufficient energy to drive cellular functions. The shifts between glycolysis, which is anaerobic and produces only a fairly small amount of ATP, and OXPHOS, which is aerobic and responsible for most of the required ATP, affect cellular activity. More specifically, bioenergetic metabolic transformations are closely correlated with functional changes in osteoblasts and immune cells.

**Figure 1:**
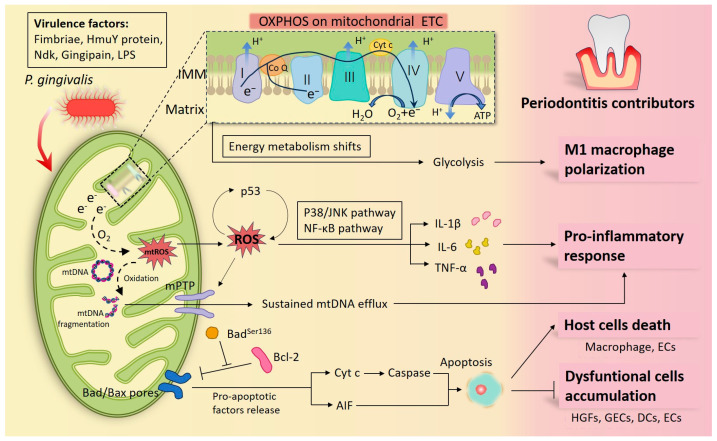
Diagram of how *Porphyromonas gingivalis* (*P. gingivalis*) participates in periodontal inflammation by interfering with mitochondria. *P. gingivalis* has multiple virulence factors that can launch an attack at the host cells. When macrophages are infected, the center of the cellular energy metabolism transfers from oxidative phosphorylation (OXPHOS) into glycolysis, and macrophages differentiate toward the pro-inflammatory M1 phenotypes. Under defective OXPHOS, abnormal electrons released from the electron transport chain (ETC) drive reactive oxygen species (ROS) formation and oxidize mitochondrial DNA (mtDNA) into fragmentation. ROS propel the release of pro-inflammatory factors by activating the P38/JNK and NF-κB signaling pathways and forming a feedback loop with p53. Additionally sustained mtDNA efflux through the ROS/mitochondrial permeability transition pore (mPTP) pathway was found under *P. gingivalis* infection, which also contributes to inflammation. In addition, plenty of studies have confirmed that by modulating mitochondria-associated apoptotic molecules, *P. gingivalis* and its virulence factors inhibited periodontal cell apoptosis, thereby prolonging periodontal inflammation, such as in human gingival fibroblasts (HGFs), gingival epithelial cells (GECs), dendritic cell (DCs), peripheral blood mononuclear cells (PBMCs), epithelial cells (ECs), etc. Nonetheless, *P. gingivalis* promoting apoptosis in a mitochondrial manner has also been documented in some host cells, such as in macrophages and ECs, as a way to indicate its toxicity. Together, these cytopathic alterations illustrate the inflammatory damage in infected periodontal tissues.

Several studies showed that macrophages and Th17 cells with OXPHOS as the main metabolic phenotype seemed to exhibit anti-periodontitis properties [47,48]. It was found that Th17 cells cultured under OXPHOS conditions are resistant to apoptotic cell death compared to those under glycolytic conditions [47]. Moreover, the conversion of pro-inflammatory M1 macrophages to anti-inflammatory M2 macrophages promoted bone formation in canine periodontal tissue defects, and this macrophage polarization change was proven to be accompanied by a shift in cellular metabolism from glycolysis and ROS generation to oxidative phosphorylation [48]. The mechanism behind the synchronization of glycolysis with increased ROS synthesis may be paralleled by the fact that, following the dominance of glycolytic ATP synthesis, the mitochondrial membrane potential (MMP) generated by the oxidative phosphorylation complexes across the IMM via proton pumping is no longer employed to drive ATP synthesis, resulting in a high MMP and increased ROS synthesis [49]. Further research confirmed that *P. gingivalis* induced a shift in macrophage metabolism from OXPHOS to glycolysis which was supported by enhanced lactate release, decreased mitochondrial oxygen consumption, and increased ROS production [50] (Figure 1). However, the mineralization of osteoblasts is dominated by glycolysis, while there is a simultaneous rise in OXPHOS and glycolysis during the initial stages of osteoblast differentiation [51,52]. Accordingly, the struggle between oxidative phosphorylation and glycolysis cannot simply be concluded as a good or bad thing, but a more important consideration is under which metabolic environment the cell can maximize its reparative function or prevent the damage caused by periodontitis.

As reputed by-products of mitochondrial energy metabolism, ROS are important condemnable mediators of *P. gingivalis*-induced periodontitis. For example, HGFs derived from patients with aggressive periodontitis exhibited higher levels of mtROS compared with those from the healthy group [53]. In vitro, LPS from *P. gingivalis* increased mtROS levels of HGFs isolated from a healthy human [54]. In addition, extensive research has verified that ROS served as key pathogenic mediators of systemic diseases, such as diabetes [55,56], atherosclerosis [57], hypertension [58], cardiovascular disease [59], benign prostatic hyperplasia [60], etc. So, it is no surprise that ROS elevation is a shared pathological link between systemic diseases and periodontitis, as *P. gingivalis* can cause oxidative stress in the arterial endothelium [61] and brain endothelial cells [62]. In parallel with affecting cellular activity, ROS also interfere with subcellular organelle functions. For instance, ROS generated from mitochondria were proven to directly modulate mitochondrial dynamics [63], mitochondrial biogenesis [64], and mitochondrial-dependent apoptosis [65]. Notably, the reduction of ROS was used as an effective indicator for periodontitis treatments, and significant alleviation and partial restorations in alveolar bone loss were observed in these trials [65,66]. Taken together, mitochondria serve as hubs for immune signaling and the generation of corresponding effectors in periodontitis, implicating the necessity of exploring the pathogenic pathway of *P. gingivalis* from the molecular level of mitochondria.

## 4. *Porphyromonas gingivalis* Causes Mitochondrial Dysfunction

### 4.1. Porphyromonas gingivalis Induces Mitochondrial Quality Control Imbalance

**Figure 2:**
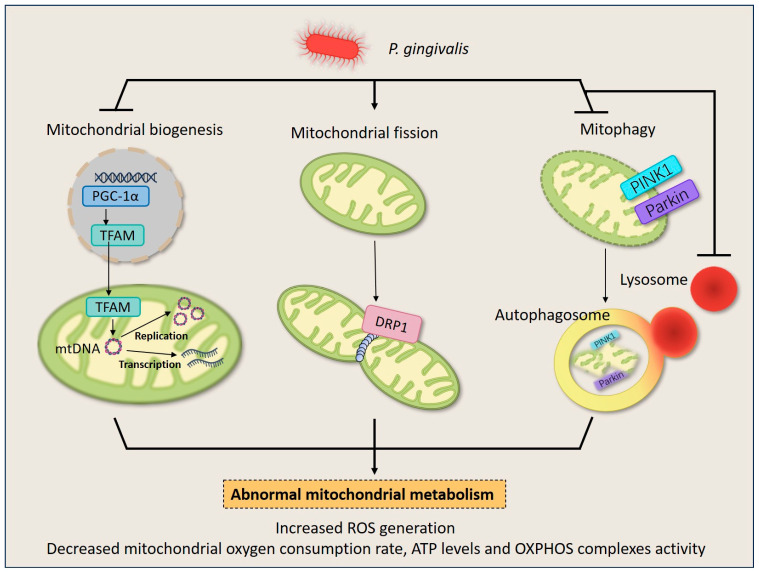
*P. gingivalis* induces mitochondrial quality control imbalance in HGFs and macrophages. Upon *P. gingivalis* infection, the life cycle of mitochondria turns into chaos. *P. gingivalis* down-regulates mitochondrial biogenesis by inhibiting the expression of PGC1-α and TFAM. It also stimulates mitochondrial fission by inducing DRP1 expression, leading to mitochondrial fragments. Additionally, *P. gingivalis* blocks mitophagy by suppressing PINK1/Parkin expression and intracellular lysosome quantity, and results in the failure of dysfunctional mitochondria elimination. The dysregulation of mitochondrial homeostasis culminates in a decrease in overall mitochondrial mass and disruption of energy metabolism, therefore deranging normal cellular behavior.

Given the main duty of mitochondria in energy production, they are exposed to high amounts of ROS making them particularly vulnerable to mtDNA mutations and protein misfolding, so they have evolved a quality control system to guarantee mitochondrial quantity and quality to match metabolic demand and maintain mitochondria homeostasis [67]. Mitochondrial quality control runs through the life cycle of mitochondria, starting with the growth and division of pre-existing organelles (mitochondrial biogenesis) and ending with the degradation of impaired or surplus organelles by mitophagy [68]. Mitochondria also undergo frequent fusion and fission events (mitochondrial dynamics) resulting in multiple scattered mitochondria or interconnected mitochondrial networks [68]. A growing wealth of evidence shows that *P. gingivalis* dysregulated mitochondrial quality control in periodontal cells, causing impaired cellular energy metabolism (Figure 2).

#### 4.1.1. *Porphyromonas gingivalis* Suppresses Mitochondrial Biogenesis and Disrupts mtDNA Homeostasis

The normal activity of cells and organelles depends on the production and interaction of specific proteins. Only a small proportion of mitochondrial proteins are encoded by the mitochondrial genome, whereas most proteins are encoded by the nuclear genome and require a coordinated import mechanism to send these proteins into the mitochondria and to assist with the eventual folding and assembly of proteins into functional complexes. So the occurrence of mitochondrial biogenesis relies on the up-regulation of transcription factors, as a way to promote sufficient mitochondrial protein production to ensure energy metabolism [69]. The major regulators include peroxisome proliferators-activated receptor γ coactivator alpha (PGC-1α), and the ultimate regulatory effector, mitochondrial transcription factor A (TFAM), which regulates the level of mtDNA replication and transcription [69]. It should be specified that though few proteins are encoded by mtDNA, they include the 13 polypeptides that constitute the ETC and a small amount of RNA required for the synthesis of mitochondrial proteins; therefore, abnormalities in mtDNA homeostasis, including aberrant mtDNA localization and copy number, as well as mtDNA damage and fragmentation, also account for mitochondrial mass deficiency [70]. There is a case to suggest that mitochondrial biogenesis disorders participate in the development of periodontitis. Miyazaki et al. confirmed that a decline in mitochondrial biogenesis mediated by TFAM deficiency led to ATP depletion and aggravated osteoclast apoptosis and bone resorption activity [71].

Studies have reported that *P. gingivalis*-LPS induced disorder of mitochondrial biogenesis and disruption of mtDNA homeostasis in HGFs [14,64,72]. It was found that *P. gingivalis*-LPS infection led to a decrease in the protein levels of phosphorylated PGC-1α and TFAM in HGFs and was accompanied by a decline in mitochondrial oxygen consumption rate and ATP levels [64]. Moreover, fewer mtDNA copy number was discovered in the *P. gingivalis*-LPS treated HGFs [14], implying that mtDNA-based protein transcriptional expression may be affected. Also, in response to *P. gingivalis*-LPS stimulation, significant efflux of mtDNA into the cytoplasm and out of the cell via a ROS/mPTP pathway was observed in primary HGFs, and the efflux lasted even in the passaged cells removed from the stimulus [72], suggesting that the spread and expansion of periodontal inflammation might be partially attributable to the persistent mtDNA efflux from infected cells and the activation of corresponding inflammatory pathways (Figure 1).

#### 4.1.2. *Porphyromonas gingivalis* Promotes Mitochondrial Fission

Within the cytoplasm, mitochondria are not all isolated organelles but also joined to form larger networks or distributed unevenly to match the local energy demands of the cell [73]. The changes in the morphology and distribution of mitochondria are mediated by fusion and fission, which are collectively referred to as mitochondrial dynamics. In mammalian cells, the fusion of mitochondria is regulated by mitofusin 1 (MFN1), MFN2, and optic atrophy protein 1, while the fission is primarily carried out by the dynamin-related protein 1 (DRP1) oligomerizing in the membrane [74]. The fragmentations brought by the over-divided mitochondria are believed to be part of the possible causes of OXPHOS failure, since deletion of mtDNA and abnormal activity of the respiratory chain complex may occur during this process [75].

*P. gingivalis* disrupts mitochondrial energy synthesis by stimulating DRP1-dependent mitochondrial fission. Experiments have confirmed that *P. gingivalis*-LPS promoted total DRP1 and phosphorylated-DRP1 (Ser616) and decreased MFN2 in mouse gingival tissue and HGFs, leading to shorter mitochondrial length and even fragmentation [54]. Furthermore, it was found that DRP1-knockdown rescued the mitochondrial fragmentation and the significant reduction in complex I activity and ATP levels brought by *P. gingivalis*-LPS infection in HGFs [76]. Moreover, the role of *P. gingivalis* on mitochondrial fission has also been verified in vascular endothelial cells, but with dissimilarities in the specific regulatory genes [77]. Specifically, up-regulation of p-DRP1 was detected, whereas total DRP1 and three fusion proteins remained unchanged [77]. Despite differences in the detection and regulation of specific mitochondrial fission and fusion proteins across studies, DRP1-mediated mitochondrial fission appears to be the main effect of *P. gingivalis*. Still, more research is needed to distinguish the effects of *P. gingivalis* and its various virulence factors on mitochondrial dynamics-related proteins in periodontal cells.

#### 4.1.3. *Porphyromonas gingivalis* Inhibits Mitophagy

Mitophagy involves distinct steps to remove defective or superfluous mitochondria, during which mitochondria are decorated with poly-ubiquitin chains, engulfed by autophagosomes, and degraded following lysosomal fusion [78,79]. So far, mitochondrial receptors including proteins containing an LIR motif (Nix, BNIP3, and FUNDC1) and the ubiquitin-relying PINK1–Parkin axis have been confirmed to be associated with mitophagy [80]. However, not all of them were proven to be differentially expressed in periodontitis. What has been documented is that *P. gingivalis* affects macrophages by restraining the classical PTEN-induced kinase 1 (PINK1)-dependent mitophagy.

Jiang et al. uncovered that the mRNA and protein levels of PINK1 and Parkin were significantly decreased in both inflammatory gingival tissues and bone marrow-derived macrophages (BMDMs) infected by *P. gingivalis* [81], suggesting that the mitophagy mechanism was impaired in periodontitis. It was subsequently revealed that mitophagy inducers dexmedetomidine, urolithin A, and resveratrol suppressed the production of IL-1β, IL-6, TNF-α, and ROS of *P. gingivalis*-triggered BMDMs; however, knockdown of PINK1 failed to have a pro-inflammatory effect [81]. These observations indicated that mitophagy inhibited by *P. gingivalis* was involved in the pro-inflammatory transformation of macrophages, but may not necessarily via a PINK1-mediated pathway.

Nevertheless, the successful activation of mitophagy also requires the coordination of lysosomes. A study demonstrated that while *P. gingivalis* promoted the expression of PINK1–Parkin in oral epithelial cells, it caused an intracellular lysosome absence and a significant reduction in the co-localization of PINK1-labeled mitochondria and lysosomes [82]. It hinted that *P. gingivalis* inhibited the ultimate degradation of dysfunctional mitochondria by posing an effect on lysosomes [82].

### 4.2. Crosstalk between Porphyromonas gingivalis and ROS

ROS are generated during mitochondrial oxidative metabolism as well as in cellular response to xenobiotics, cytokines, and bacterial invasion. Although the main ROS production comes from mitochondria, organelles like peroxisomal and endoplasmic reticular could also produce ROS [83]. ROS includes oxygen ions/O_2_^•^, free radicals (superoxide/O^2−^ and hydroxyl radicals), and peroxides (hydrogen peroxide/H_2_O_2_) [84]. At the very beginning, ROS was simply thought to be a kind of toxic by-product in an aerobic environment, but gradually more studies proved their specific role as intracellular signaling molecules contributing to retrograde redox signaling from the organelle to the cytosol and nucleus, regulating a variety of homeostatic cellular functions [85,86]. ROS can activate signaling pathways to initiate biological processes in physiological and pathological conditions [87]. More remarkably, when ROS production overwhelms the cellular antioxidant defense system, oxidative stress occurs [88]. Oxidative stress has long been regarded as the pathological environment of periodontitis. *P. gingivalis* contributes to the formation of an oxidative stress environment in periodontitis by increasing mtROS production.

Mitochondria are the major source of intracellular ROS, playing a role as an important intermediate mediator of *P. gingivalis*-activated inflammation [89]. A study revealed that the increase in the concentration of mtROS preceded the expression of inflammatory cytokines in HGFs under *P. gingivalis*-LPS treatment, and further experiments demonstrated that elimination of mtROS successfully suppressed the LPS-induced p38/JNK signaling pathway and NF-κB signaling pathway, thus decreasing the expression of IL-1β, IL-6, and TNF-α [89]. Studies further characterized that the increase and mitochondrial translocation in p53 formed a feedback loop with ROS, together participating in the inflammatory response above [90,91] (Figure 1). Moreover, while *P. gingivalis* triggers inflammation by inducing mtROS, mtROS also act as central pathogenic factors evoking dysfunction and aberrant activity of cells. Bullón revealed that mtROS modulate cellular activity in *P. gingivalis*-LPS infected HGFs by down-regulating mitochondrial biogenesis and contributing to cell death [64]. Nonetheless, whether the elevated mtROS are caused by intact *P. gingivalis* or by certain virulence factors remains elusive. For example, Liu et al. proved the supernatant of *P. gingivalis* did not induce ROS in human oral epithelial cells, but the invasion of the intact *P. gingivalis* did induce ROS [82].

Although *P. gingivalis* appears to trigger oxidative stress by stimulating an increase in ROS, causing periodontal damage, ROS themselves own an antibacterial effect. Generally, ROS generation can be applied by host cells as a defensive mechanism to combat intracellular pathogens. West et al. identified that macrophages generated mtROS to resist bacterial invasion through the direct communication of activated TLRs with mitochondrial Complex I [92]. However, *P. gingivalis* seems to have some specificity in avoiding oxidative damage. *P. gingivalis* can sense the oxidative stress in the environment and potentiate its gene expression concerned with antioxidant regulators, including OxyR and RprY [93]. And studies have proved that the haem layer on the surface of *P. gingivalis* serves as a buffer layer against oxidative attacks [94,95]. Moreover, these antioxidant responses of *P. gingivalis* were further amplified in plaque biofilms [96,97].

The causes of the oxidative stress state in the periodontal microenvironment may be comprehensive and not limited to *P. gingivalis*-induced ROS generation. Thus, the role of *P. gingivalis* on host cells in the context of oxidative stress seemed to be quite complicated and somehow controversial. On the one hand, oxidative stress aggravates the aggressiveness of *P. gingivalis* against host cells. Knowles et al. revealed that gingipain exacerbated the suppression of transcriptional function in oral epithelial cells under oxidative stress [98]. And macrophage endotoxin tolerance to *P. gingivalis*-LPS was hindered under an oxidative stress environment, therefore contributing to periodontitis progression [99]. On the other hand, *P. gingivalis* also restrains cellular exogenous oxidative stress to some extent, prolonging its pathogenic effect. It was found that when gingival epithelial cells (GECs) underwent oxidative stress in response to extracellular ATP stimulation, *P. gingivalis*-Nucleoside-diphosphate-kinase (Ndk) infection up-regulated the antioxidant glutathione and inhibited cytosolic and mitochondrial ROS generation thus blocking the oxidative stress and maintaining its persistence intracellularly [100].

Intriguingly, based on the dual pro-inflammatory and antimicrobial effects of ROS, new treatments have emerged to suppress periodontal bacteria and improve cellular status by appropriately promoting oxidative stress in the periodontal microenvironment. Photobiomodulation, an emerging periodontal treatment, converts cellular metabolism into mitochondrial OXPHOS and enhances cellular antioxidant activity by inducing a modest boost in ROS [101]. Evidence has demonstrated that treating periodontitis tissue of beagles with photobiomodulation decreased the concentration of all dominant pathogens, including *P. gingivalis*, *Fusobacterium nucleatum*, and *Corynebacterium* [102].

### 4.3. Porphyromonas gingivalis Influences Mitochondria-Mediated Apoptosis

Cell apoptosis is initiated by extracellular and intracellular signals via two main pathways, the death receptors and the mitochondria-mediated pathways [103]. The collapse of mitochondrial inner transmembrane potential and mitochondrial outer membrane permeabilization (MOMP) play a primary and central role in mitochondria-mediated apoptosis, causing uncontrolled mitochondrial energy metabolism, activation, and spillover of mitochondrial pro-apoptotic effectors [104,105]. There are a host of mitochondrial pro-apoptotic factors presented in mitochondria and released into the cytoplasm upon induction of apoptosis, including cyt c, apoptosis inducing factor (AIF), Smac/DIABLO, and several procaspases (such as procaspase-2, -3, and -9) [106]. It is worth noting that cyt c release is one of the most common and persuasive indicators of mitochondria-mediated apoptosis, whereas relatively few studies have been conducted on the AIF-release-determined pathway. AIF is unique in that when released as a pro-apoptotic factor, it translocates directly to the nucleus and mediates DNA fragmentation through the binding of nucleic acid endonucleases, distinguishing itself from the caspase-dependent apoptosis pathways mediated by cyt c release and death receptor [107,108]. The effect of *P. gingivalis* on mitochondria-mediated apoptosis is inconsistent in terms of different cell types and virulence factors (Figure 1).

It was reported *P. gingivalis* suppressed mitochondria-mediated apoptosis. Bax/Bak and Bcl-2-like proteins, when activated, are translocated from the cytosol to the OMM, where they undergo conformational rearrangement and assembly to regulate the MOMP and the release of mitochondrial-apoptotic mediators [42,109]. By down-regulating the expression of pro-apoptotic protein Bax and up-regulating the anti-apoptotic protein Bcl-2, *P. gingivalis* tended to keep the cells active at 24 h [110]. Moreover, subsequent studies confirmed that *P. gingivalis* inhibited mitochondria-mediated apoptosis of primary GECs through the activation of the phosphatidylinositol 3-kinase/Akt pathway which mediated the up-regulation of the phosphorylated Bad^Ser136^ preventing the pro-apoptotic protein Bad from competitively binding to Bax against Bcl-2 [111,112,113]. Similarly, Desta et al. found pretreatment of HGFs with *P. gingivalis* attenuated TNF-α-stimulated apoptosis, and when AIF was down-regulated by siRNA, cell death was reduced dramatically [114].

Nevertheless, studies on the role played by *P. gingivalis*-specific virulence factors in the resistance of mitochondria-mediated apoptosis have also been inconsistent in both periodontal and non-periodontal cells. Though, it was demonstrated that *P. gingivalis* similarly suppressed dendritic cell apoptosis by promoting Bcl-2, the different fimbriae phenotypes (i.e., fimA and Mfa1) exemplified marked differences in their regulatory roles [115]. Intriguingly, Mao et al. pointed out that the anti-apoptotic activity by *P. gingivalis* in hGECs did not require the presence of fimbriae [116]. In addition, *P. gingivalis* HmuY protein, which helps *P. gingivalis* uptake heme for nutrition, delays apoptosis in peripheral blood mononuclear cells via a Bcl-2-regulated way, thereby prolonging the release of cellular inflammatory factors [117,118]. As in non-periodontal cells, Lee et al. demonstrated that *P. gingivalis*-Ndk inhibited mitochondria-mediated apoptosis in adipocytes by directly binding to and phosphorylating the Heat-shock-protein-27 and the ndk-deficient *P. gingivalis* did not ameliorate staurosporine-induced apoptosis, showing the irreplaceable role of *P. gingivalis*-ndk [119]. Surprisingly, Boisvert et al. inhibited staurosporine-induced apoptosis in laryngeal cancer epithelial cells simply with purified gingipain adhesin peptide A44, which may be related to cell specificity [120]. In conclusion, the anti-apoptotic manifestation of *P. gingivalis* benefits its intracellular survival and prolongs the inflammatory state brought by infected cells.

Even with copious studies above showing that *P. gingivalis* inhibited apoptosis in the mitochondrial pathway, the phenomenon of mitochondria-associated apoptosis caused by *P. gingivalis* has also been observed in some cells. THP-1 macrophages showed a shift in mitochondria-associated apoptotic proteins towards pro-apoptosis in response to *P. gingivalis* stimulation [121]. In addition, several studies demonstrated that butyric acid, metabolites of *P. gingivalis*, caused mitochondria-dependent apoptosis in HGFs, but the ultimate nuclear DNA damage severity and apoptosis sensitivity correlated with the duration and concentration of butyric acid, and inflammatory environment [122,123]. Likewise, the apoptotic effect of butyric acid has also been observed in nerve cells, which may account for neuropathic pain in the varying stages of periodontitis [124]. It has also been shown that *P. gingivalis* induced apoptosis in epithelial cells through the AIF release pathway [125]. Moreover, mitochondria-dependent apoptosis under *P. gingivalis* was also detected in tissues outside the periodontal correlation, including cardiomyocytes [126], vascular endothelial cells [127], adipocytes [128], and skin fibroblasts [129]. The initiation of mitochondria-mediated apoptosis by *P. gingivalis* reflects more of its dual effects in causing a total collapse of cellular structure and function while suppressing chronic bacterial infection accumulation.

## 5. Conclusions

The mitochondrial pathway is an inextricable link in the pathology of periodontitis due to *P. gingivalis*. It causes inflammatory destruction of periodontal tissues and bone resorption by invading intracellularly, penetrating the epithelial barrier, and attacking fibroblasts and immune cells in the deeper tissues. Under certain circumstances, *P. gingivalis* enters the blood circulation to influence the development of systemic diseases. Mitochondrial homeostasis has an imperative role in the periodontal environment.

In summary, *P. gingivalis* triggers an imbalance in mitochondrial quality control, leading to an accumulation of defective mitochondria, and a sustained mtROS and efflux of DAMPs which trigger inflammatory responses. Moreover, *P. gingivalis* owns anti-mitochondrial-mediated apoptotic properties, therefore maintaining the local accumulation of pro-inflammatory cells. However, the anti-apoptotic effects of *P. gingivalis* were not presented in all cells, and the molecular mechanisms underlying the mitochondrial dysfunction caused by *P. gingivalis* have not been fully investigated. Additionally, studies on how to ameliorate *P. gingivalis*-associated periodontitis by intervening with mitochondria will be instrumental in further optimizing mitochondria-targeted periodontitis therapy.
